# Microfluidics-Assisted Polymer Vesicle Budding in Emulsion Systems: A Promising Approach for the Preparation and Application of Polymer Vesicles

**DOI:** 10.3390/molecules29204802

**Published:** 2024-10-11

**Authors:** Donghua Dong, Jilai Zhan, Guoxing Liao, Tong Zhu, Qianqian Yu, Wei Zhang, Linge Wang

**Affiliations:** South China Advanced Institute for Soft Matter Science and Technology, School of Emergent Soft Matter, Guangdong Provincial Key Laboratory of Functional and Intelligent Hybrid Materials and Devices, Guangdong Basic Research Center of Excellence for Energy & Information Polymer Materials, South China University of Technology, Guangzhou 510640, China; ddhhard@163.com (D.D.); jilai_zhan@163.com (J.Z.); gxliao@scut.edu.cn (G.L.); 202310192955@scut.edu.cn (T.Z.)

**Keywords:** microfluidic, emulsion template, polymer vesicle formation, vesicle budding dynamics

## Abstract

The challenge of producing polymer vesicles remains difficult, despite numerous attempts to modulate the kinetics of polymer vesicle budding and achieve precise control over the membrane characteristics. An innovative approach that incorporates the use of copolymer-loaded single-emulsion droplets is proposed to address this challenge. This method enables the precise manipulation of micelles and polymer vesicles’ composition, structures and dimensions. The emulsion contracts and forms microspheres when the copolymer concentrations exceed > 0.5 wt%, resulting in the formation of nano polymer vesicles. Conversely, the copolymer spontaneously forms micro polymer vesicles and micelles through vesicle budding at lower concentrations. The spontaneous production of vesicles and micelles can be induced by modifying the copolymer concentration in the emulsion. Our discoveries have a significant impact relative to the development of copolymer membranes and contribute to an enhanced comprehension of the mass manufacturing of polymer vesicles from single emulsions.

## 1. Introduction

Polymer vesicles (or polymersomes) are self-assembled shells of amphiphilic block copolymers (BCPs) which have garnered significant interest for their ability to efficiently encapsulate and deliver therapeutic agents (such as drugs and proteins) intracellularly [1,2]. Polymer vesicles have found wide-ranging applications in biomedical contexts and in the life sciences, including use as gene carriers [3,4], artificial cell membranes [5,6], nanoreactors [7,8,9], pesticides [10] and drug delivery systems [11,12,13,14,15]. Several methods have been employed to prepare polymer vesicles in aqueous solutions, including film-hydration [16,17,18,19], solvent exchange [2,20] and electroformation [21,22]. The energetic penalty during the formation of spherical vesicles from the membrane does not depend on the radius, but rather on the sum of the mean and Gaussian curvature. Consequently, solvent exchange techniques typically lack a robust size-selection mechanism [2,23]. Furthermore, the unbinding process in the film-hydration procedure is contingent upon variations in the membrane. A robust and reliable method for the preparation of polymeric vesicles is emerging as a significant potential advancement in the field. Hence, the utilization of photolithography [21,22], sonification [24,25] and extrusion [26,27] have been employed to control the size of polymer vesicles.

**Scheme 1:**
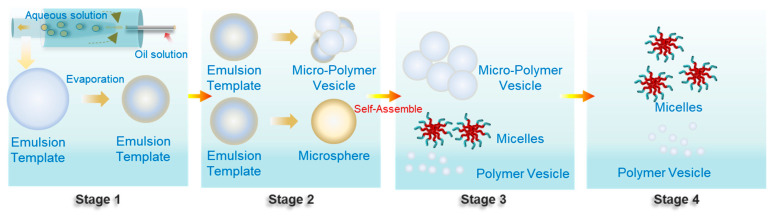
Emulsion templates and polymer vesicle production from emulsion templates.

Microfluidics is also a technology that is intended to produce monodispersed micro-scale emulsion droplets and streams that can be used to construct double layers of polymer vesicles [28,29,30,31]. Recently several microfluidic technologies have been applied for the preparation of polymer vesicles, including the double emulsion [29,30,32], droplet emulsion transfer [33], jet-flow [34] and pulsed-jetting [35], and flow-focusing methods [36,37]. The double emulsion template is a typical method to prepare polymer vesicles, but, due to the limitation of micro-scale channels in the microfluidic system, only micro-scale polymer vesicles can be fabricated by this method [5,38]. Therefore, mechanical division and extrusion methods have been launched to produce both micro- and nano-scale polymer vesicle [28,39]. Despite the notable progress made in these methodologies, their intricate nature and restricted capacity for expansion continue to pose substantial obstacles in the context of large-scale production [40]. Drawing inspiration from the phenomenon of vesicle budding observed in double-emulsion templates and micro-scale copolymer patches, this study introduces a novel approach wherein polymer vesicles are generated spontaneously from single emulsions. Similar to the precise control achieved in lithography, microparticles, and double-emulsion templates derived from copolymer patches, it permits the precise adjustment of the size and membrane properties of our polymer vesicles through the utilization of droplet microfluidics, as illustrated in Scheme 1. The proposed methodology offers a direct and efficient technique for the synthesis of polymer vesicles, budding from a single-emulsion template. The present study utilized microfluidics techniques to achieve the fabrication of polymer vesicles by employing single-emulsion templates. Due to its biocompatibility and amphiphilicity, a BCP, Poly(ethylene glycol)-*b*-Poly(lactic acid) (PEG-*b*-PLA), serves as copolymer material and surfactant. Monodisperse emulsions were generated via co-flow microfluidic devices with different concentrations. Several imaging techniques, including Confocal Light Scanning Microscopy (CLSM), Scanning Electron Microscope (SEM), Optical microscopy (OM), and Transmission Electron Microscope (TEM), were utilized to study the progression of this transformation process at the microscopic level. The empirical investigation revealed that the process of self-assembly from single-emulsion templates is contingent upon the concentration of PEG-*b*-PLA.

## 2. Results and Discussion

### 2.1. Preparation of Emulsion Templates

**Figure 1:**
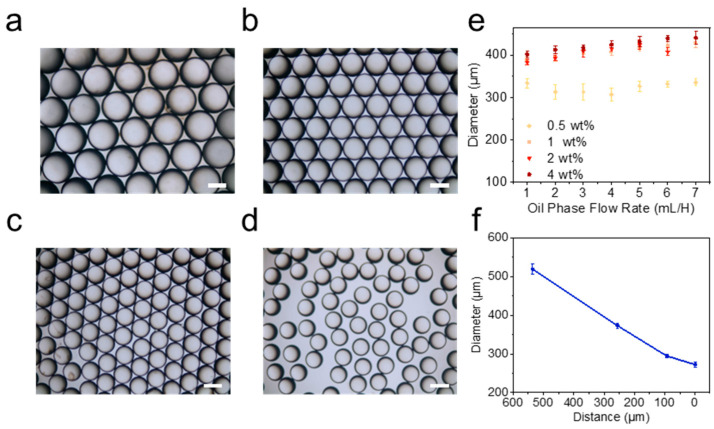
OM images of O/W single emulsions and statistical data associated with the diameters of the emulsion templates. (**a**–**d**) OM images of a single-emulsion template with different reception distances between the inlet capillary and glass needle: 530 µm, 256 µm, 93 µm and 0 µm, respectively. The concentration of the PVA aqueous solution is 0.5 wt%, and the scale bar is 300 µm. (**e**) Statistical data associated with the emulsion template diameter with different oil flow rates and concentrations. (**f**) Statistical data associated with emulsion template diameter with different collect diameters.

The selection of PEG_113_-*b*-PLA_167_ for producing polymer vesicles was based on its biocompatibility and degradability. Microfluidics is widely used to prepare emulsions, liposomes and polymer vesicles, due to its exceptional ability to control liquid phases precisely. The present investigations involve the design of a resilient microfluidic device (Figure S1) consisting of a metal needle, glass capillary and connectors. An metal needle and a glass capillary are aligned to achieve tip-to-tip alignment. This microfluidic device can be assembled modularly with considerable simplicity. Emulsion templates were made via the microfluidic device. Diameters of the emulsion ranged from 250 µm to 500 µm as shown (Figure 1 and Figure S2) with low coefficients of variation (CVs) (below 5%), indicating excellent size controllability. Analyzing the CLSM images shows that the emulsion template exhibits uniform size (Figure S3).

Due to the concentration-dependent nature of polymer vesicles, as previously established in refs [41,42], the emulsion template has been specifically formulated to encompass a range of PEG_113_-*b*-PLA_167_ concentrations, including 0.03 wt%, 0.04 wt%, 0.25 wt%, 0.5 wt%, 2.0 wt% and 4.0 wt%, in sequential order. Analysis of OM image indicates that PEG_113_-*b*-PLA_167_ concentration does not significantly influence the initial diameter of the emulsion templates. The flow rates for the oil phase and the aqueous solution flow rate were set as 3.0 mL/h and 30.0 mL/h, respectively, due to no significant emulsion template size changes when varying flow rates (Figure 1e and Figure S2).

### 2.2. Self-Assembly and Polymer Vesicle Preparation

**Figure 2:**
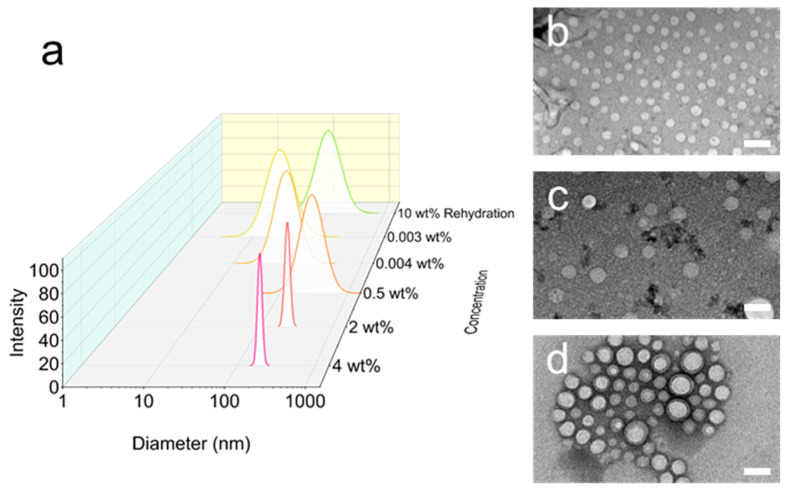
Morphologies and diameter distribution of PEG_113_-*b*-PLA_167_ self-assembly after evaporation for 24 h. (**a**) Diameter distribution of PEG_113_-*b*-PLA_167_ self-assembly in PVA aqueous solution and PEG_113_-*b*-PLA_167_ polymer vesicles made from film rehydration. (**b**–**d**) TEM images of self-assembly of PEG_113_-*b*-PLA_167_ prepared by emulsion template. The scale bar is 100 nm.

PEG_113_-*b*-PLA_167_ emulsion templates were produced at various concentrations, ranging from 0.03 wt% to 4.0 wt%. The TEM image revealed that within a concentration range of 0.03 wt% from 4.0 wt%, the self-assembly morphology transforms from micelles (20–35 nm) to polymer vesicles, with diameters ranging from 120 to 150 nm (Figure 2b–d). And the PDI of polymeric vesicles (PDI 0.06) prepared by the emulsion template method is significantly smaller than that of the film rehydration method (PDI 0.20). The mass yield of vesicle production was 3–300 times higher compared to other methodologies used for polymer vesicle production (Table S1). To ensure self-assembly at a concentration of 0.03 wt%, the fluorescent indicator Nile Red was used to determine the critical micelles concentration (CMC) of the PEG_113_-*b*-PLA_167_. The result revealed that the CMC was 50 µg/mL (Figure S4), which corresponds to the mass concentration of the 0.03 wt% PEG_113_-*b*-PLA_167_ emulsion template in aqueous solution (Equation (S1)). These results suggested that the assemblies observed at this particular concentration were micelles. However, based on the DLS data (Figure 2a), it can be determined that at low concentrations (<0.25 wt%), the PDI of self-assembly is broader than at 4 wt% (Figure S5). The results indicate a wider variety of assemblies within the solution at low concentrations.

**Figure 3:**
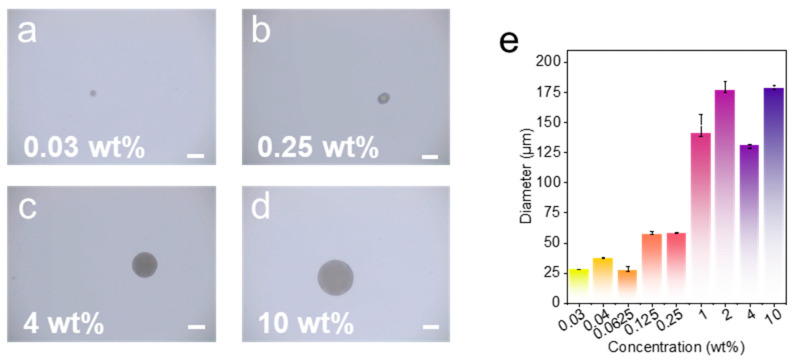
Optical microscopy and diameter of emulsion templates with different BCP concentrations after evaporation. (**a**–**d**) Optical microscope images of emulsion templates with different concentrations: 0.03 wt%, 0.25 wt%, 4.0 wt% and 10.0 wt%, respectively. Scale bar is 300 µm. (**e**) Diameters of the emulsion templates after evaporation.

**Figure 4:**
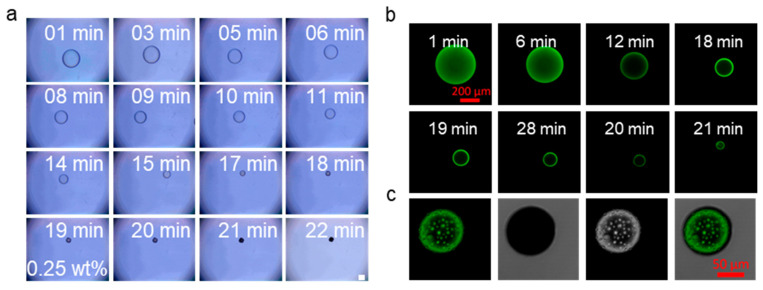
Optical and CLSM images of PEG_113_-*b*-PLA_167_ O/W single-emulsion templates. (**a**) Optical microscopy images of PEG_113_-*b*-PLA_167_ O/W single-emulsion droplets at different evaporation times. The origin picture is represented in (Figure S7). The scale bar is 300 µm. (**b**,**c**) CLSM of PEG_113_-*b*-PLA_167_ O/W single-emulsion template at different evaporation times and micro-scale polymer vesicle buds from emulsion. (**c**) Enlarged view of (**b**) at 21 min. The initial concentration of PEG_113_-*b*-PLA_167_ is 0.25 wt%. The fluorescent agent is Cour-6.

The evaporation process of emulsion templates was investigated to elucidate various self-assembly distributions. It was found that the emulsion templates undergo a comprehensive transformation into microspheres upon evaporation without any extra interference (Figure 3). By adjusting the concentration of the BCP, the observed range of final template diameters ranges from 25 µm to 200 µm (Figure 3). Notably, it was found that 0.25 wt% is a critical concentration, at which BCP experienced swelling and micro-size polymer vesicles budded on the surface of the emulsion template (Figure 3b). The DCM exhibits a higher evaporation rate for the emulsion’s outer edges than in the central region (Figure S6). Therefore, a single-emulsion droplet was extracted from the plate and subsequently studied using OM and CLSM (Figure 4b).

### 2.3. Evaporation and Self-Assembly in Aqueous Solution

Optical imaging was employed at various time intervals to monitor the morphological changes in a single droplet of the emulsion template during the evaporation process. The results revealed a steady reduction in the size of the emulsion over time (Figure 4a). As the diameter of the emulsion template decreased, the BCP showed swelling, and the micro-scale polymer vesicles budded at a diameter range of 10-25 µm (Figure 4). After 12 h evaporation, the micro-scale polymer vesicles were gradually diffused into the solution (Figure S8) and observed through OM and SEM. A more pronounced development of budding resulted, which was more clearly seen in the CLSM after the introduction of Cour-6 into the oil solution and micro-scale polymer vesicles (Figure 4b). This implies that low concentrations facilitate the production of vesicle outgrowths on a micrometer scale.

**Figure 5:**
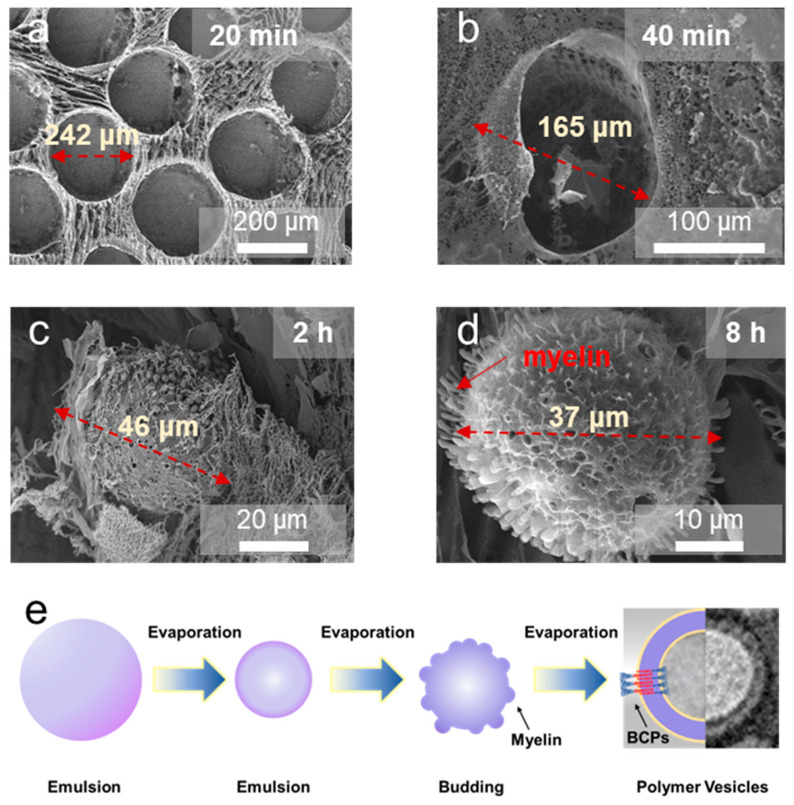
Cryo-SEM images of the emulsion during evaporation. (**a**–**d**) Cryo-SEM images of emulsion at 20 min, 40 min, 2 h and 8 h, respectively. The concentration of PEG_113_-*b*-PLA_167_ emulsion is 0.5 wt%. (**e**) Schematic diagram of emulsion outgrowth to form vesicles.

Interestingly, an alternative phenomenon was observed with concentrations higher than 0.50 wt%. In such cases, the emulsion shrank into a microsphere, and the budding process for micro polymer vesicles did not occur. Subsequently, nano polymer vesicles were observed to form during the 2 h of evaporation (Figure 5). The cross-section diameter of the myelin on the surface of single-emulsion templates, known as metastable structures [43], was 640 ± 160 nm (Figure 5d). Concurrently, the scattering light photon count rate in the solution also increased as the evaporation progressed, suggesting a continuous diffusion of nano polymer vesicles from the emulsion (Figure S9). Based on the observations presented, the budding process from the surface of the polymer vesicles was conceptualized as the evagination of the copolymer membrane. In the initial step, BCPs aggregate at the surface of the emulsion, as illustrated in (Figure 5a). In the subsequent step, additional BCPs accumulate on the emulsion surface as the emulsion size decreases. Concurrently, as the organic phase diffuses into the aqueous phase, the original hydrophobic block aggregates form a new polymer block and contract under surface tension. Since the swelling of the polymer vesicle membranes by water invasion, the surplus copolymer molecules within the swollen membranes gain mobility and self-assemble into a new copolymer bilayer through lateral diffusion. Regarding BCP concentrations exceeding 0.25 wt%, it is essential to note that a higher concentration of BCP in the emulsion template can potentially restrict the mobility of BCPs. This is because BCPs have a very low CMC and slow chain exchange, indicating that they form locally isolated, nonergodic structures [44]. As a result, amphiphile diffusion occurs not as individual molecules but as the collective diffusion of these nonergodic assemblies [45,46]. In the first step, BCPs aggregate on the surface of the emulsion. The introduction of water leads to the swelling of the upper layer of the copolymer. At low polymer concentrations (equal to or below 0.25 wt%), the diffusion of water molecules leads to the formation of micro-scale vesicles. Conversely, at high concentrations (above 0.5 wt%), organic-solvent residues fail to plasticize the dense emulsion. Thereby restricting free diffusion within the rigid capsule and hindering the development of larger copolymer membranes. This finding provides novel perspectives on the process of polymer vesicle formation. The results indicate that the formation of nano polymer vesicles can occur, via the budding process, from an emulsion template instead of just self-assembling directly from micelles. This phenomenon is different from the process Julian et al. initially observed in the formation of microscopic vesicle budding from double-emulsion templates [47]. Compared with previous research [47], this work now confirms that the occurrence of nano-scale vesicle budding strongly depends on BCPs concentration and relates to the evaporation process.

**Figure 6:**
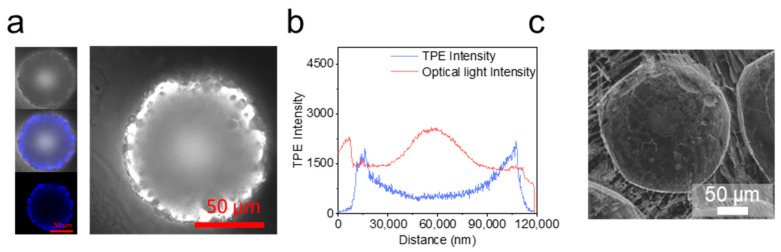
Morphology and structure of PEG_113_-*b*-PLA_167_ O/W single-emulsion templates. The concentration of PEG_113_-*b*-PLA_167_ emulsion is 0.25 wt%. (**a**) CLSM images of PEG_113_-*b*-PLA_167_ O/W single-emulsion droplets with TPE. (**b**) Cross-section of the fluorescent intensity of the TPE in the emulsion at the times denoted in (**a**). (**c**) Cryo-SEM images of PEG_113_-*b*-PLA_167_ O/W single-emulsion droplets after 1 min of evaporating.

To further prove this phenomenon, an aggregation-induced emission (AIE) molecule known as TPE and exhibiting distinct AIE behavior was introduced into the oil solution. TPE derivatives have been found to exhibit torsional and rotational motions, showing distinct AIE activity [48]. This phenomenon is associated with the self-assembly process of BCP in aqueous solution, which is driven by the hydrophobic effect [49,50,51]. Based on Zhang’s work, the evolution of the fluorescence intensity of TPE in polymer vesicles can be used to distinguish two major stages in the self-assembly process [49]. From the CLSM image (Figure 5a), fluorescence intensity within the emulsion template, extending from the center towards the periphery, indicates a probable preferential aggregation of polymer molecules at the emulsion’s edge. This aggregation likely leads to restricted mobility of the TPE molecules, thereby producing regions with heightened fluorescence. The fluorescence profile of the single emulsion template matches the morphology and structure recorded by Cryo-SEM (Figure 6). The findings of this experiment imply that, at the low concentrations of the experimental samples, BCPs tend to aggregate preferentially at the surface of the emulsion template, leading to the formation of a BCPs film. The results indicate that the molecular concentration within the emulsion does not maintain a homogeneous distribution during the period in which the emulsion evaporates.

The Cryo-SEM technique was employed to better understand the internal mechanisms behind the emulsion’s evaporation process. Cryo-SEM study revealed a decrease in the diameter of the emulsion in the initial stages due to evaporation. Additionally, a thin layer was observed forming on the emulsion’s surface (Figure S10). The following expression allows the calculation of PEG_113_-*b*-PLA_167_ *Rg* [52,53,54]:

(1) $${Rg}^{2}=\frac{b^{2}*{\left(\frac{lc}{b}\right)}^{2\nu }}{\left(2\nu +1\right)*(2\nu +2)}$$

where b is the statistical segment length and N is the degree of polymerization. The ν is scaling, and lc is chain segment length. The 2*Rg of PEG_113_-*b*-PLA_167_ is 10.55 nm. So the thin layer of PEG_113_-*b*-PLA_167_ can be seen as a double-layer or six-layer membrane, which is close to the thickness of polymer vesicles.

Based on the information above, it can be inferred that the evaporation of the organic solvent from the emulsion droplet leads to a reduction in the size of the polymer-containing droplet. This process eventually results in ordered polymer structural domains forming near the interface. During subsequent solvent evaporation, an aqueous solution invades this molecular layer, forming micro polymer vesicles. Further solvent evaporation results in the breakdown and fragmentation of micro-scale polymer vesicles within the aqueous solution, forming micelles and polymer vesicles. Although the concentration of PEG_113_-*b*-PLA_167_ exceeds 0.5 wt%, the freezing and gradual rehydration of BCPs in an aqueous environment can be attributed to solvent evaporation. Finally, nano polymer vesicles bud from the microsphere.

Notably, the sizes of the polymer vesicles formed from several BCPs, including PEG_56_-*b*-PCL_66_, PEG_113_-*b*-PCL_105_ and PEG_91_-*b*-PLA_111_, were closely comparable (Figure S11). This indicates that the methodology yields comparable preparative outcomes across various polymer molecules, thereby showcasing its potential applicability.

### 2.4. Drug Loading and Cell Biocompatibility

**Figure 7:**
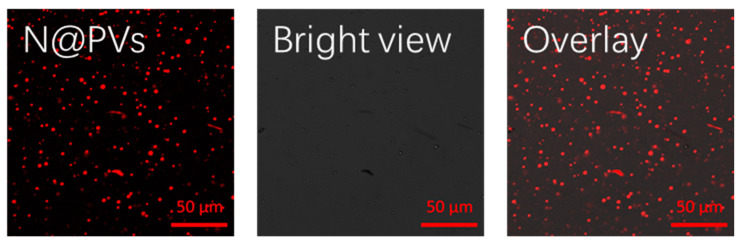
CLSM images of polymer vesicles containing Nile Red. N@PVs = polymer vesicles containing Nile Red.

To evaluate the drug loading efficiency of the polymer vesicles that were produced, DOX and Nile Red were used as model drugs. The drug loading efficiency ranged from 5.3 wt% to 10.0 wt%. The zeta potential of the polymer vesicles exhibited a consistent range from −5.0 to −15.0 mV (Figure S12). CLSM images of the polymer vesicles confirmed the effective loading of Nile Red into the respective polymer vesicles (Figure 7). The results for drug loading efficiency were not significantly different from those of the conventional solvent exchange method. Therefore, further research is needed to improve the drug loading.

4T1 cells were cultured on 96-well plates with Nile Red polymer vesicles and pure polymer vesicles for 12 h and 24 h. Based on the CCK-8 assay (Figure S13), 4T1 had an excellent survival rate, and viability remained at 80% when co-incubated with polymer vesicles. The results of the CLSM image analysis indicate that cells can produce Nile Red polymer vesicles, suggesting that the vesicle has the potential for biological applications (Figure S13b).

## 3. Experimental Design

### 3.1. Materials

Two PEG-*b*-PLA samples, i.e., PEG_113_-*b*-PLA_167_ (*M*_w_ = 18,200 g/mol, *Đ*(*M*_w_/*M*_n_) = 1.20) and PEG_91_-*b*-PLA_111_ (*M*_w_ = 10,300 g/mol, *Đ*(*M*_w_/*M*_n_) = 1.40); two Poly (ethylene glycol)-Poly(caprolactone) (PEG-*b*-PCL) samples, i.e., PEG_56_-*b*-PCL_66_ (*M*_w_ *=* 10,500 g/mol, *M*_w_/*M*_n_ 1.08) and PEG_113_-*b*-PCL_105_ (*M*_w_ *=* 12,500 g/mol, *Đ*(*M*_w_/*M*_n_) = 1.08); and one Poly (ethylene glycol)-*b*-Poly(styrene) sample (PEG-*b*-PS), PEG_45_-*b*-PS_50_ (*M*_w_ *=* 7200 g/mol, *Đ(M*_w_/*M*_n_) = 1.30), were purchased from Jinan Dai gang Biomaterial Co., Ltd.(Jinan, China). *M*_n_ and *M*_w_ are the number- and weight-averaged molecular weights. Methylene chloride (DCM) was obtained from Sigma-Aldrich (St. Louis, MO, USA). Polyvinyl alcohol (PVA, molecular weight range of 22,000~24,000 g/mol) was purchased from a commercial supplier, Shanghai Yingjia Co., Ltd. (Shanghai, China). Deionized water was produced using a water purification system (Shanghai, China). Coumarin-6 (Cour-6) was obtained from Aladdin Industrial Corporation (Shanghai, China). Chloroform (purity ≥ 99%), Tetraphenylethylene (TPE) and Doxorubicin Hydrochloride (DOX·HCl) were purchased from Aladdin Industrial Corporation (Shanghai, China). The calcein acetoxymethyl ester (calcein-AM), Dulbecco’s modified Eagle medium (DMEM), Fetal bovine serum (FBS) and propidium iodide (PI) were sourced from the Beyotime Institute of Biotechnology in China. Unless otherwise stated, all of the chemicals were employed as received.

### 3.2. Microfluidic Equipment

The device in this study consists of two cylindrical capillaries coaxially arranged to achieve tip-to-tip alignment. The inlet capillary is made of steel and has a diameter of 100 µm. The continuous phase is delivered through a 300 µm glass needle.

### 3.3. Single-Emulsion Template Preparation

The copolymer DCM solution (0.03–10 wt%) is a dispersed phase (oil phase) pumped into the inlet, with the flow rate ranging from 1 mL/h to 7 mL/h, respectively. Polyvinyl alcohol (PVA) aqueous solution (0.5 wt%) is pumped from outlets as the continuous phase, with the flow rate ranging from 10 mL/h to 70 mL/h, respectively. The distance between the inlet capillary and the collect glass needle ranges from tip-to-tip to 500 µm.

### 3.4. Sample Preparation and Aggregation Studies

Self-assembly and polymer vesicles are made from a single-emulsion template after solvent evaporation for 24 h (stirring speed 100 rpm, room temperature). After evaporation, the solution was centrifugally separated by 1000 rpm, and the supernatant then taken.

The CMC studies of PEG_113_-*b*-PLA_167_ follow the method reported by Raju Bej et al. [55]. In all, 500 µL of PBS buffer (pH approximately 7.4) was added to various vials to create a series of solutions, maintaining a constant NR concentration of 10.0 µM and adjusting the polymer concentrations between 0.1 and 500.0 µg/mL. Each solution was subjected to sonication for 15 min and then allowed to equilibrate for 2 h. The emission spectra for the NR probe were measured using Spectra Max iDx (Molecular Devices, San Jose, CA, USA).

### 3.5. Characterization of Emulsion Templates and Polymer Vesicles

The emulsion templates and polymer vesicles were observed using OM and CLSM (Zeiss, LSM 880 NLO, Oberkochen, Germany) in PVA solution, for which process a droplet was placed in a 10 mL PVA solution in a surface dish. The diameter distribution of the emulsion was measured by ImageJ 1.48V software. Cour-6 and TPE were added to the oil phase for the observation of the volatilization process of the single-emulsion templates. Representative SEM images of emulsion templates and polymer vesicles were accomplished by SEM (JEOL, JSM-7900F, Tokyo, Japan). To enhance the signal quality, samples were sputtered by platinum for 3 min, operating at an accelerating voltage of 5 kV. In Cryo-SEM, samples were frozen in liquid nitrogen, then sublimated for 30 min, and gold spraying was effectuated for 3 min. Representative TEM images of polymer vesicles were characterized using TEM (JEOL, JEM-1400 Flash, Tokyo, Japan) after staining with 2 wt% sodium phosphotungstate. The self-assembly of block copolymers was analyzed by dynamic light scattering instrument (DLS, Brookhaven, Omni, NY, USA) for size, polydispersity index (PDI), and zeta potential. The test angle was 90 degrees, and the temperature was 25 °C.

### 3.6. Drug Loading Studies

To demonstrate the method for drug delivery, Nile Red and DOX were utilized as hydrophobic and hydrophilic drugs, respectively, and encapsulated within the polymer vesicles via the single-emulsion method. Nile Red and DOX were dissolved in oil phase solvents and then the emulsion template and polymer vesicles were prepared. The final product was dialyzed under constant stirring for 48 h in DI water.

The drug loading efficiency (DLE) was calculated by following Equation (2).

(2) $$DLE\left(\%\right)=\frac{W_{Load}}{W_{Total}}\times 100\%$$

W_load_ is the loading weight of polymer vesicles and W_total_ is the total weight of DOX and Nile Red. Nile Red and DOX concentrations were measured by UV-vis (Shimadzu, UV-3600Plus, Kyoto, Japan) and a fluorescence spectrophotometer (HITACHI F-4600, Tokyo, Japan).

### 3.7. Cell Culture and Real-Time Living Cell Imaging

The 4T1 cells, at a density of 1.0 × 10^3^ cells per well, were seeded in 96-well plates and cultured with DMEM supplemented with 10% FBS, penicillin and streptomycin, under a humidified atmosphere. The viability and proliferation of 4T1 cells cultured on different polymer vesicle samples were evaluated using the CCK-8 assay. The optical density (OD) was recorded, and the hemolytic ratio was calculated as follows:

(3) $$Hemolytice\;ratio=\frac{OD_{t}-OD_{N}}{OD_{P}-{OD}_{N}}\times 100\%$$

Here, OD_n_, OD_p_, and OD_t_ were the absorbance values of samples, negative control (PBS) and positive control (water), respectively.

Real-time observation of the cellular uptake and localization of polymer vesicle delivery systems was accomplished by CLSM. Cells were cultured at different intervals in fresh medium containing pure polymer vesicles and Nile Red-labeled polymer vesicles at 37 °C. CLSM images were analyzed using AxioVision 4.2 software (Carl Zeiss, White Plains, NY, USA).

## 4. Conclusions

A microfluidic technology was utilized to synthesize the emulsion template for preparing polymer vesicles. It reveals that polymer concentration is an essential factor in the formation of polymer vesicles budding. Regions of high concentration are prone to the formation of narrowly dispersed nano-scale vesicles, while micro-scale vesicles and micelles tend to arise at lower concentrations. This result suggests a unique intermediate process for preparation of polymer vesicles using the emulsion template method, which diverges significantly from the conventional double-emulsion template method [44]. Research also investigations on cell cytophagy have demonstrated that the vesicles show favorable biocompatibility, offer potential for various biological applications. 

## Data Availability

Data are contained within the article and Supplementary Materials.

## Supplementary Materials

The following supporting information can be downloaded at: https://www.mdpi.com/article/10.3390/molecules29204802/s1. Refs [55,56] are cited in the Supplementary Materials.

## References

[1] Discher B.M., Won Y.-Y., Ege D.S., Lee J.C.-M., Bates F.S., Discher D.E., Hammer D.A. (1999). Polymersomes: Tough vesicles made from diblock copolymers. Science.

[2] Discher D.E., Eisenberg A. (2002). Polymer vesicles. Science.

[3] Christian D.A., Cai S., Bowen D.M., Kim Y., Pajerowski J.D., Discher D.E. (2009). Polymersome carriers: From self-assembly to siRNA and protein therapeutics. Eur. J. Pharm. Biopharm..

[4] Zou Y., Zheng M., Yang W., Meng F., Miyata K., Kim H.J., Kataoka K., Zhong Z. (2017). Virus-Mimicking Chimaeric Polymersomes Boost Targeted Cancer siRNA Therapy In Vivo. Adv. Mater..

[5] Fanalista F., Birnie A., Maan R., Burla F., Charles K., Pawlik G., Deshpande S., Koenderink G.H., Dogterom M., Dekker C. (2019). Shape and Size Control of Artificial Cells for Bottom-Up Biology. ACS Nano.

[6] Houbrechts M., Caire da Silva L., Ethirajan A., Landfester K. (2021). Formation of giant polymer vesicles by simple double emulsification using block copolymers as the sole surfactant. Soft Matter.

[7] Che H.L., van Hest J.C.M. (2019). Adaptive Polymersome Nanoreactors. ChemNanoMat.

[8] Vriezema D.M., Garcia P.M., Sancho Oltra N., Hatzakis N.S., Kuiper S.M., Nolte R.J., Rowan A.E., Van Hest J.C. (2007). Positional assembly of enzymes in polymersome nanoreactors for cascade reactions. Angew. Chem.-Int. Ed..

[9] Che H., Cao S., van Hest J.C.M. (2018). Feedback-Induced Temporal Control of “Breathing” Polymersomes to Create Self-Adaptive Nanoreactors. J. Am. Chem. Soc..

[10] Xiao W., Cao X., Yao P., Garamus V.M., Chen Q., Cheng J., Zou A. (2022). Enhanced Insecticidal Effect and Interface Behavior of Nicotine Hydrochloride Solution by a Vesicle Surfactant. Molecules.

[11] Ke W., Li J., Mohammed F., Wang Y., Tou K., Liu X., Wen P., Kinoh H., Anraku Y., Chen H. (2019). Therapeutic Polymersome Nanoreactors with Tumor-Specific Activable Cascade Reactions for Cooperative Cancer Therapy. ACS Nano.

[12] Li W.-P., Su C.-H., Chang Y.-C., Lin Y.-J., Yeh C.-S. (2016). Ultrasound-Induced Reactive Oxygen Species Mediated Therapy and Imaging Using a Fenton Reaction Activable Polymersome. ACS Nano.

[13] Wang S., Liu Y., Xu M., Hu F., Yu Q., Wang L. (2022). Polymersomes as virus-surrogate particles for evaluating the performance of air filter mate-rials. Giant.

[14] Kala C., Asif M., Gilani S.J., Imam S.S., Khan N.A., Taleuzzaman M., Zafar A., Ahmed M.M., Alshehri S., Ghoneim M.M. (2022). Formulation of Isopropyl Isothiocyanate Loaded Nano Vesicles Delivery Systems: In Vitro Characterization and In Vivo Assessment. Molecules.

[15] Li W., Liu S., Yao H., Liao G., Si Z., Gong X., Ren L., Wang L. (2017). Microparticle templating as a route to nanoscale polymer vesicles with controlled size distribution for anticancer drug delivery. J. Colloid Interface Sci..

[16] Battaglia G., Ryan A.J. (2005). Bilayers and interdigitation in block copolymer vesicles. J. Am. Chem. Soc..

[17] Kita-Tokarczyk K., Grumelard J., Haefele T., Meier W. (2005). Block copolymer vesicles—Using concepts from polymer chemistry to mimic biomembranes. Polymer.

[18] Reeves J.P., Dowben R.M. (1969). Formation and properties of thin-walled phospholipid vesicles. J. Cell. Physiol..

[19] Contini C., Pearson R., Wang L., Messager L., Gaitzsch J., Rizzello L., Ruiz-Perez L., Battaglia G. (2018). Bottom-Up Evolution of Vesicles from Disks to High-Genus Polymersomes. iScience.

[20] Soo P.L., Eisenberg A. (2004). Preparation of block copolymer vesicles in solution. J. Polym. Sci. Part B-Polym. Phys..

[21] Le Berre M., Yamada A., Reck L., Chen Y., Baigl D. (2008). Electroformation of giant phospholipid vesicles on a silicon substrate: Advantages of controllable surface properties. Langmuir.

[22] Howse J.R., Jones R.A., Battaglia G., Ducker R.E., Leggett G.J., Ryan A.J. (2009). Templated formation of giant polymer vesicles with controlled size distributions. Nat. Mater..

[23] Bergström M., Eriksson J.C. (1998). Size distribution of reversibly formed bilayer vesicles. Langmuir.

[24] Pereira-Lachataignerais J., Pons R., Panizza P., Courbin L., Rouch J., Lopez O. (2006). Study and formation of vesicle systems with low polydispersity index by ultrasound method. Chem. Phys. Lipids.

[25] Chen W., Du J. (2013). Ultrasound and pH Dually Responsive Polymer Vesicles for Anticancer Drug Delivery. Sci. Rep..

[26] Frisken B.J., Asman C., Patty P.J. (2000). Studies of Vesicle Extrusion. Langmuir.

[27] Rusli W., van Herk A.M. (2021). Effect of salts on size and morphology of extruded dimethyldioctadecylammonium bromide or chloride vesicle for polymeric nanocapsules synthesis via templating emulsion polymerization. J. Colloid Interface Sci..

[28] Zhang H., Cui W., Qu X., Wu H., Qu L., Zhang X., Mäkilä E., Salonen J., Zhu Y., Yang Z. (2019). Photothermal-responsive nanosized hybrid polymersome as versatile therapeutics codelivery nanovehicle for effective tumor suppression. Proc. Natl. Acad. Sci. USA.

[29] Utada A.S., Lorenceau E., Link D.R., Kaplan P.D., Stone H.A., Weitz D.A. (2005). Monodisperse double emulsions generated from a microcapillary device. Science.

[30] Shum H.C., Kim J.-W., Weitz D.A. (2008). Microfluidic fabrication of monodisperse biocompatible and biodegradable polymersomes with controlled permeability. J. Am. Chem. Soc..

[31] Nguyen X.D., Park D.H., Paik H.-J., Jeon H.J., Huh J., Go J.S. (2020). Microfluidic Tracking of the Growth of Polymeric Vesicles in Hydrodynamic Flow. ACS Appl. Polym. Mater..

[32] Shum H.C., Santanach-Carreras E., Kim J.-W., Ehrlicher A., Bibette J., Weitz D.A. (2011). Dewetting-Induced Membrane Formation by Adhesion of Amphiphile-Laden Interfaces. J. Am. Chem. Soc..

[33] Matosevic S., Paegel B.M. (2011). Stepwise Synthesis of Giant Unilamellar Vesicles on a Microfluidic Assembly Line. J. Am. Chem. Soc..

[34] Kamiya K., Kawano R., Osaki T., Akiyoshi K., Takeuchi S. (2016). Cell-sized asymmetric lipid vesicles facilitate the investigation of asymmetric membranes. Nat. Chem..

[35] Stachowiak J.C., Richmond D.L., Li T.H., Brochard-Wyart F., Fletcher D.A. (2009). Inkjet formation of unilamellar lipid vesicles for cell-like encapsulation. Lab Chip.

[36] Thiele J., Steinhauser D., Pfohl T., Förster S. (2010). Preparation of Monodisperse Block Copolymer Vesicles via Flow Focusing in Mi-crofluidics. Langmuir.

[37] Jahn A., Vreeland W.N., Gaitan M., Locascio L.E. (2004). Controlled vesicle self-assembly in microfluidic channels with hydrodynamic focusing. J. Am. Chem. Soc..

[38] Deshpande S., Dekker C. (2018). On-chip microfluidic production of cell-sized liposomes. Nat. Protoc..

[39] Deshpande S., Spoelstra W.K., van Doorn M., Kerssemakers J., Dekker C. (2018). Mechanical Division of Cell-Sized Liposomes. ACS Nano.

[40] Zhu Y., Yang B., Chen S., Du J. (2017). Polymer vesicles: Mechanism, preparation, application, and responsive behavior. Prog. Polym. Sci..

[41] Bej R., Achazi K., Haag R., Ghosh S. (2020). Polymersome Formation by Amphiphilic Polyglycerol-*b*-polydisulfide-*b*-polyglycerol and Glutathione-Triggered Intracellular Drug Delivery. Biomacromolecules.

[42] Shen H.W., Eisenberg A. (1999). Morphological phase diagram for a ternary system of block copolymer PS310-b-PAA(52)/dioxane/H2O. J. Phys. Chem. B.

[43] Battaglia G., Ryan A.J. (2006). Neuron-like tubular membranes made of diblock copolymer amphiphiles. Angew. Chem.-Int. Ed..

[44] Battaglia G., Ryan A.J. (2005). The evolution of vesicles from bulk lamellar gels. Nat. Mater..

[45] Julicher F., Lipowsky R. (1993). Domain-induced budding of vesicles. Phys. Rev. Lett..

[46] Jain S., Bates F.S. (2004). Consequences of nonergodicity in aqueous binary PEO−PB micellar dispersions. Macromolecules.

[47] Thiele J., Chokkalingam V., Ma S., Wilson D.A., Huck W.T.S. (2014). Vesicle budding from polymersomes templated by microfluidically prepared double emulsions. Mater. Horiz..

[48] Yang Z., Qin W., Leung N.L.C., Arseneault M., Lam J.W.Y., Liang G., Sung H.H.Y., Williams I.D., Tang B.Z. (2016). A mechanistic study of AIE processes of TPE luminogens: Intramolecular rotation vs. configurational isomerization. J. Mater. Chem. C.

[49] Zhang N., Chen H., Fan Y., Zhou L., Trépout S., Guo J., Li M.-H. (2018). Fluorescent Polymersomes with Aggregation-Induced Emission. ACS Nano.

[50] Guan W., Zhou W., Lu C., Tang B.Z. (2015). Synthesis and Design of Aggregation-Induced Emission Surfactants: Direct Observation of Micelle Transitions and Microemulsion Droplets. Angew. Chem. Int. Ed..

[51] Chen H., Li M.-H. (2019). Recent Progress in Fluorescent Vesicles with Aggregation-induced Emission. Chin. J. Polym. Sci..

[52] Liao G., Chen L., Zhang Y., Mykhaylyk O.O., Topham P.D., Toolan D.T., Derry M.J., Howse J.R., Yu Q., Feng G. (2023). Solvent selectivity governed self-assembly of block copolymer in nanofabrication. Polymer.

[53] Dinic J., Sharma V. (2020). Flexibility, Extensibility, and Ratio of Kuhn Length to Packing Length Govern the Pinching Dynamics, Coil-Stretch Transition, and Rheology of Polymer Solutions. Macromolecules.

[54] Cheng S., Kogut D., Zheng J., Patil S., Yang F., Lu W. (2024). Dynamics of polylactic acid under ultrafine nanoconfinement: The collective interface effect and the spatial gradient. J. Chem. Phys..

[55] He J., Wang L., Wei Z., Yang Y., Wang C., Han X., Nie Z. (2013). Vesicular Self-Assembly of Colloidal Amphiphiles in Microfluidics. ACS Appl. Mater. Interfaces.

[56] Pecora R. (2000). Dynamic light scattering measurement of nanometer particles in liquids. J. Nanopart. Res..

