# Supplementary material for: Microfluidics-Assisted Polymer Vesicle Budding in Emulsion Systems: A Promising Approach for the Preparation and Application of Polymer Vesicles
Source: Molecules. 2024 Oct 11;29(20):4802. doi: 10.3390/molecules29204802 (PMC11510250; doi:10.3390/molecules29204802)
Supplement: Supplementary file 1 [file molecules-29-04802-s001.zip › molecules-3208313-supplementary.pdf]

Supplementary Information

Microfluidic-Assisted Polymer vesicles Budding in Emulsion Systems: a Promising Approach for Polymer Vesicles Preparation and Application

Donghua Dong[1], JiLai Zhan[1], Guoxing Liao[1], Tong Zhu[1], Qianqian Yu[1,*], Wei Zhang[1,*], LinGe Wang[1,*]

[1] South China Advanced Institute for Soft Matter Science and Technology, School of Emergent Soft Matter, Guangdong Provincial Key Laboratory of Functional and Intelligent Hybrid Materials and Devices, Guangdong Basic Research Center of Excellence for Energy & Information Polymer Materials, South China University of Technology, Guangzhou 510640, China

*Corresponding authors: yuqianqian@scut.edu.cn (Q. Q. Yu), weizhang@scut.edu.cn (W. Zhang), lingewang@scut.edu.cn (L. G. Wang)

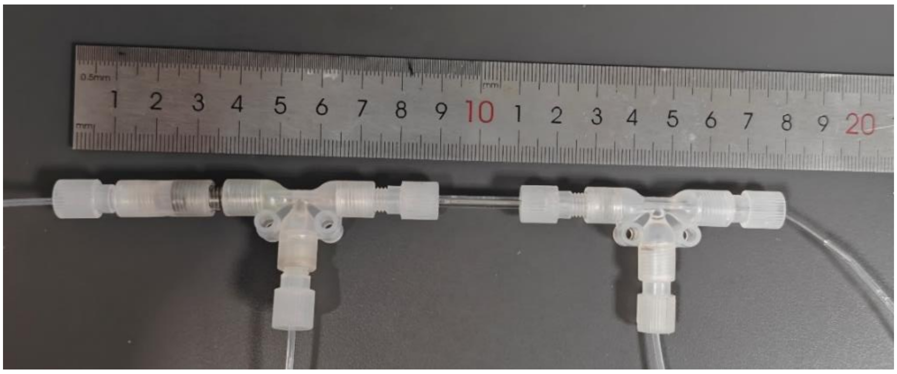

Figure S1. Digital graph of microfluid system. Microfluidic device consists of a metal needle with a thread and connectors, which can coaxially arrange to achieve tip-to-tip alignment quickly.

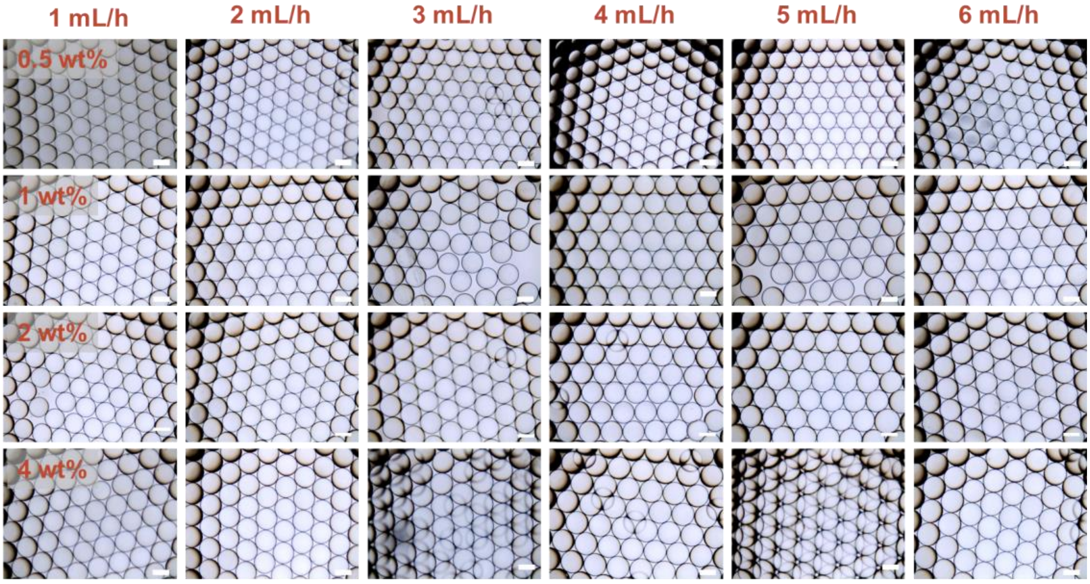

Figure S2. Optical images of emulsion with different concentrations and oil phase rates. All scale bar are 300 μm.

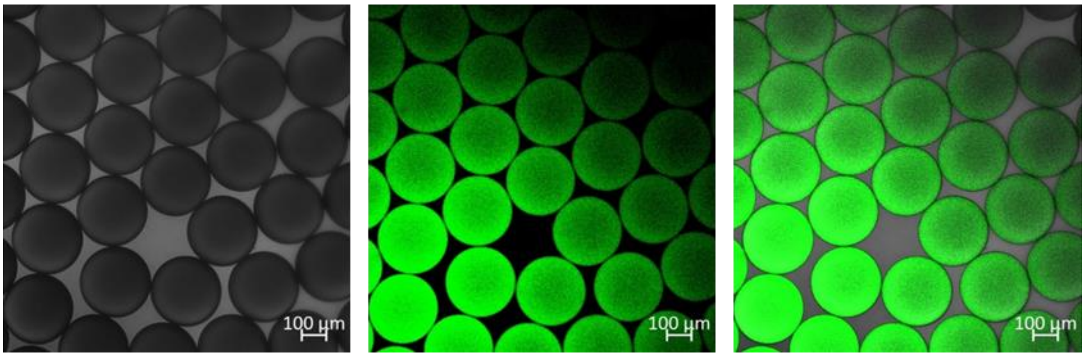

Figure S3. Confocal laser scanning microscope images of PEG_{113}-*b*-PLA_{167} O/W single emulsion template.

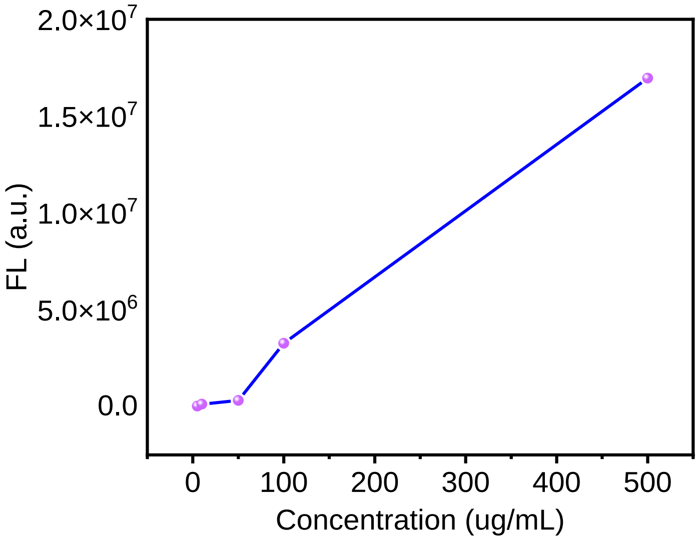

Figure S4. The plot of emission intensity (λ_{em} = 618 nm) as a function of the concentration of PEG_{113}-*b*-PLA_{167}. (Nile Red concentration is kept constant as 1.0 μM).

Equation S1-S3 Weight concentration of BCP, calculate as follow:

$$C_{\mathrm{F}} = \frac{C_{\mathrm{BCP}} \times V_{\text{flow rate of oil phase}} \times \rho \times T}{V_{\text{flow rate of aqueous solution phase}} \times T} \qquad \text{Equation (S1)}$$

C_{BCP} is the mass concentration of BCP in the solution, $V_{\text{flow rate of oil phase}}$ is flow rate of BCP solution, $V_{\text{flow rate of aqueous phase}}$ is flow rate of 0.5 wt% PVA solution, T is time, and ρ is density of organic soluiton. For DCM, ρ is equal to 1.32 g/cm^3). In the 0.03 wt% sample, the low rates of BCP solution (oil phase) and PVA solution (aqueous phase) are 5 mL/h and 30 mL/h. After one hour of collection, emulsion templates were placed in a stirring stand at room temperature (24 °C) for 24 h. The aqueous phase is only slightly volatilized during this process, so the volume reduction is negligible.

$$C_{\mathrm{F}} = \frac{0.03\ \text{wt\%} \times 5\ \frac{\mathrm{mL}}{\mathrm{h}} \times \frac{1.32\ \mathrm{g}}{\mathrm{cm}^3} \times 1\ \mathrm{h}}{35\ \frac{\mathrm{mL}}{\mathrm{h}} \times 1\ \mathrm{h}} \qquad \text{Equation (S2)}$$

$$C_{\mathrm{F}} = 66\ \mu\mathrm{g/mL} \qquad \text{Equation (S3)}$$

The mass yield of this methodology is 370 mg/h. (BCPs 4 wt%, flow rate of oil solution 3 mL/h). Mass yield compared with different methodology is list in fellow table.

Table-S1 Mass Yield Comparison

Methodology	Mass Yield	Times
Flow Focusing[1, 2]	0.45 mg/h, 11 mg/ h	33-820
Double Emulsion Templates[3, 4]	11 mg/ h，3 mg/ h	33-123
Phase Transfer[5]	9 mg/ h	>40
Double Emulsion Budding[6]	120 mg/ h	>3
Single Emulsion Template	370 mg/ h	1

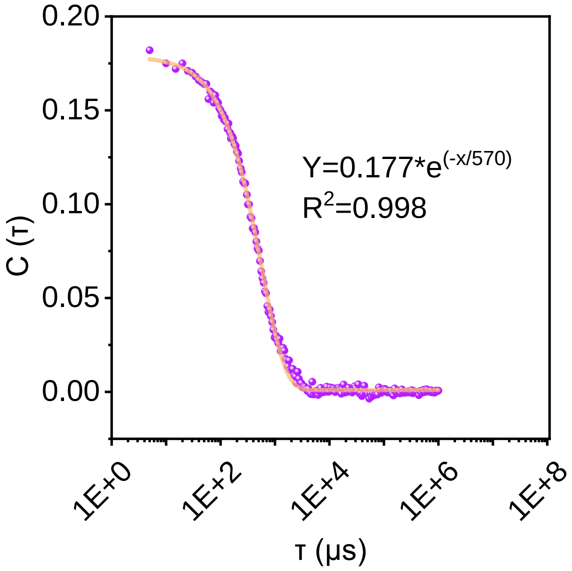

Figure S5. Delay time function ($C(\tau)$) and its fitting function of PEG$_{113}$-*b*-PLA$_{167}$ polymer vesicles. Exponential fitting results show that the curve is a monoexponential function. It indicated polymer vesicles is monodispersity in the solution.[7]

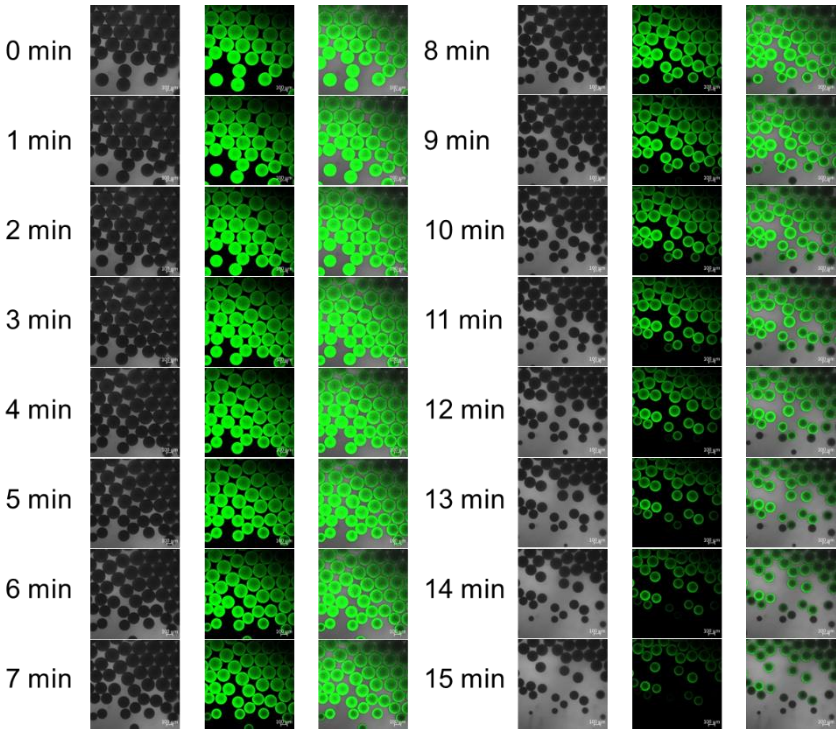

Figure S6. Emulsion evaporates process of PEG$_{113}$-*b*-PLA$_{167}$ single emulsion template. (PEG$_{113}$-*b*-PLA$_{167}$ concentration is 0.5 wt%)

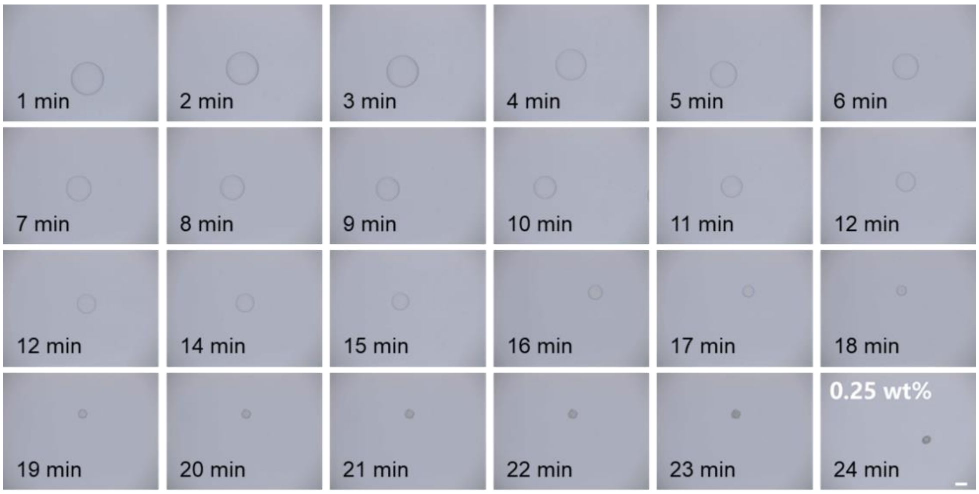

Figure S7. Optical microscopy images of PEG_{113}-*b*-PLA_{167} O/W single emulsion droplets during evaporation process. Scale bar is 300 μm. The initial concentration of PEG_{113}-*b*-PLA_{167} is 0.25 wt%.

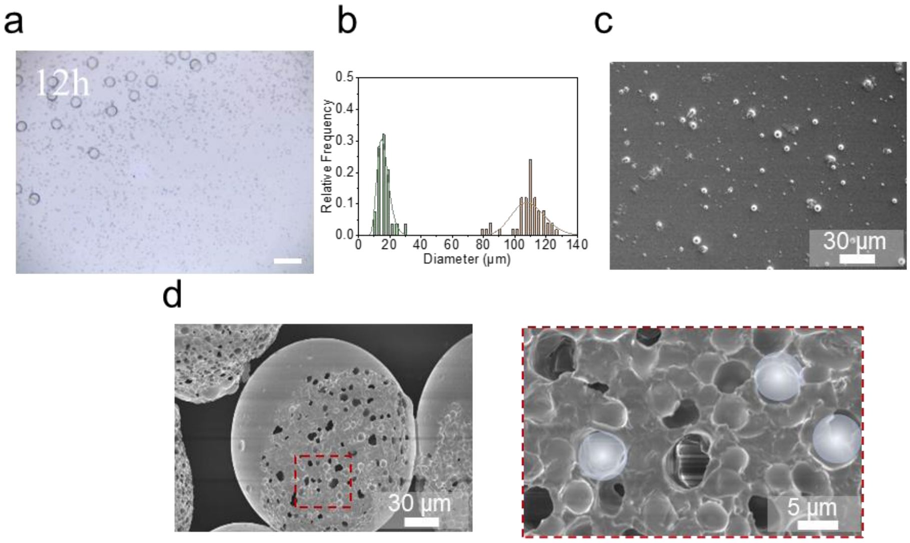

Figure S8. Optical microscope image and SEM image of PEG_{113}-*b*-PLA_{167} micro-sized polymer vesicles, PEG_{113}-*b*-PLA_{167} concentration is 0.25 wt%. (a) Optical image of isolated polymer vesicles. (b) statistical data on isolated diameter of micro-sized polymer vesicles and single emulsion template in the optical microscope image after evaporating 12 h. (c) SEM image of PEG_{113}-*b*-PLA_{167} micro-sized polymer vesicles. (d) SEM image of PEG_{113}-*b*-PLA_{167} microsphere after emulsion evaporated 24 h with the enlarged section in red box.

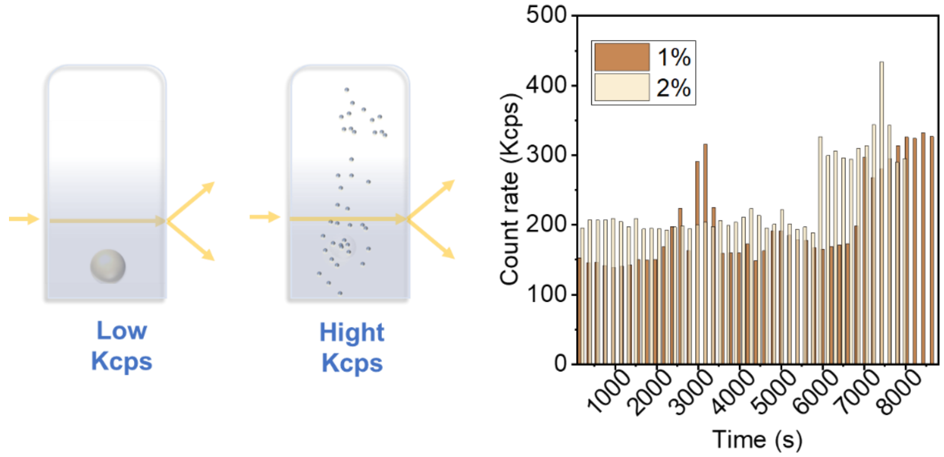

Figure S9. Schematic of emulsion (BCPs concentration are 1 wt% and 2wt%) via evaporation measured by DLS and kilo count rate (Kcps) in 2 mL 0.5 wt% PVA solution cell.

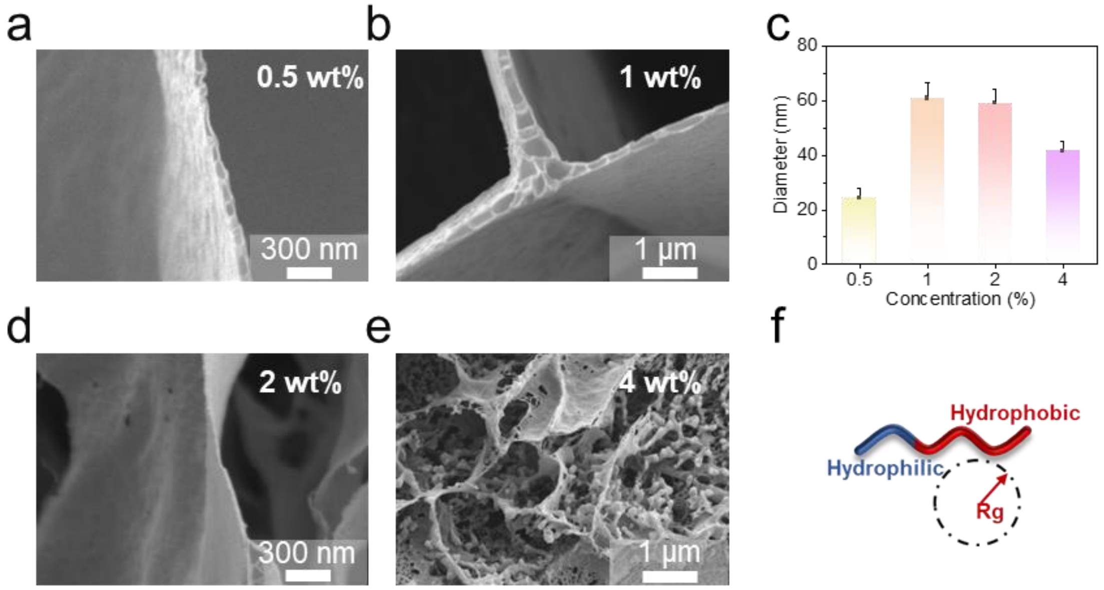

Figure S10. Cryo-SEM images of emulsion in 0.5 wt% PVA solution at 0 min. (a-e) Membrane and membrane thickness within the emulsion with the concentration of PEG_{113}-*b*-PLA_{167} are 0.5 wt%, 1wt%, 2wt%, 4wt%, respectively. (f) Schematic of diblock copolymer.

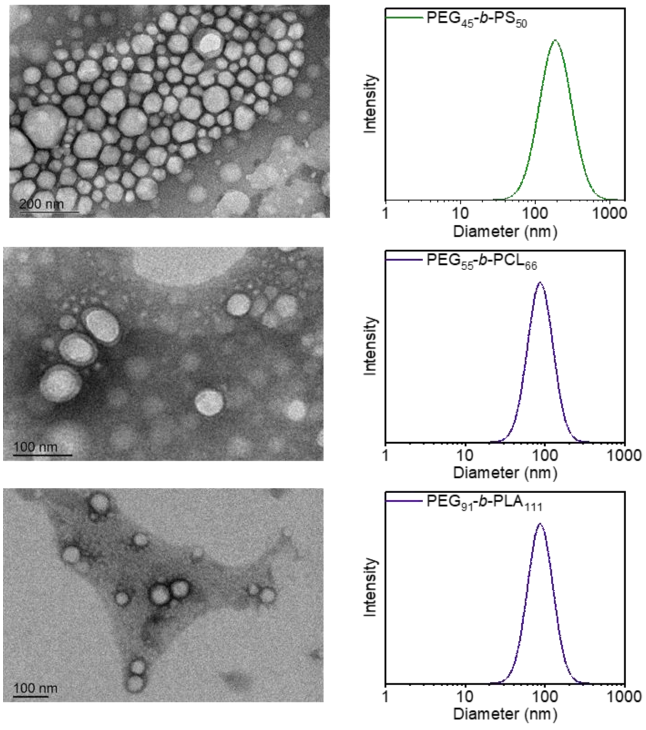

Figure S11. TEM images and size distributions of self-assembled particles of PEG_{45}-*b*-PS_{50} PEG_{56}-*b*-PCL_{66}, PEG_{91}-*b*-PLA_{111}, respectively.

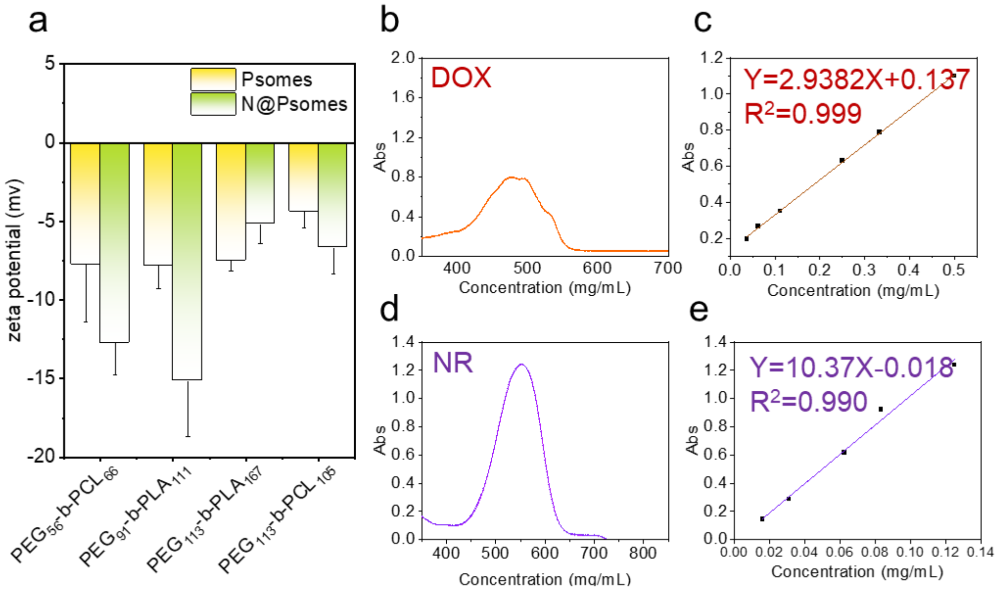

Figure S12. Zeta potential and UV absorption of polymer vesicles and N@Psomes. (a) Zeta potential polymer vesicles. (b-d) UV absorption curve and standard curve of Nile Red and dox solution respectively. Psomes= polymer vesicles, N@Psomes= polymer vesicles containing Nile Red.

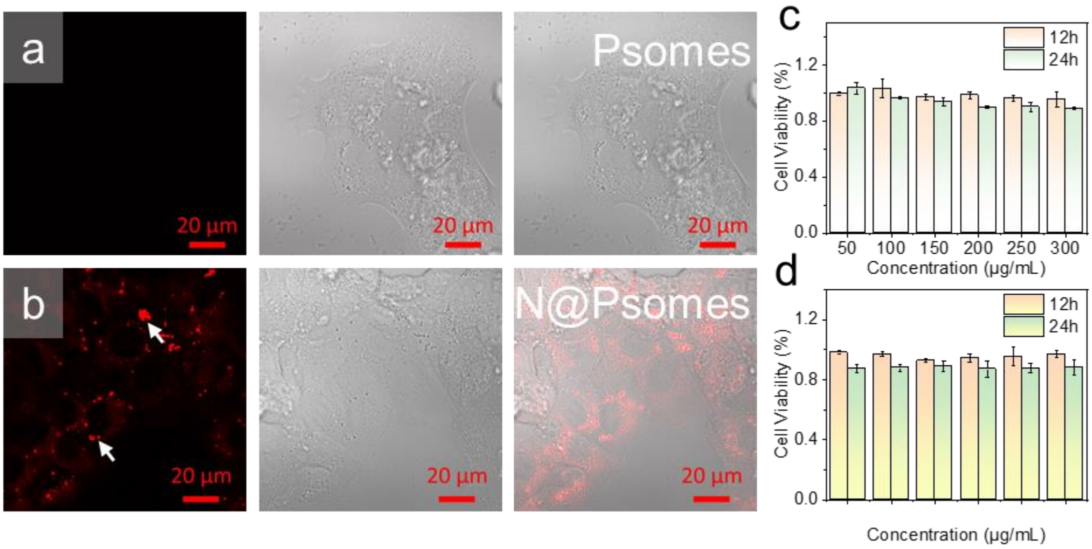

Figure 13. (a,b) Confocal fluorescence microscopy images of polymer vesicles after co-incubating in 4T1 cells in 24 h. (c,d) cell viability of polymer vesicles incubated in 12 h and 24 h.

Reference

1. Thiele, J.; Steinhauser, D.; Pfohl, T., et al. Preparation of Monodisperse Block Copolymer Vesicles via Flow Focusing in Microfluidics. *Langmuir* 2010**,** 26, 6860-6863

2. He, J.; Wang, L.; Wei, Z., et al. Vesicular Self-Assembly of Colloidal Amphiphiles in Microfluidics. *ACS Appl. Mater. Interfaces* 2013**,** 5, 9746-9751

3. Zhang, H.; Cui, W.; Qu, X., et al. Photothermal-responsive nanosized hybrid polymersome as versatile therapeutics codelivery nanovehicle for effective tumor suppression. *PNAS* 2019**,** 116, 7744-7749

4. Shum, H. C.; Kim, J.-W.; Weitz, D. A. Microfluidic fabrication of monodisperse biocompatible and biodegradable polymersomes with controlled permeability. *J. Am. Chem. Soc.* 2008**,** 130, 9543-9549

5. Matosevic, S.; Paegel, B. M. Stepwise Synthesis of Giant Unilamellar Vesicles on a Microfluidic Assembly Line. *J. Am. Chem. Soc.* 2011**,** 133, 2798-2800

6. Thiele, J.; Chokkalingam, V.; Ma, S., et al. Vesicle budding from polymersomes templated by microfluidically prepared double emulsions. *Mater. Horiz.* 2014**,** 1, 96-101

7. Pecora, R. Dynamic light scattering measurement of nanometer particles in liquids. *J. Nanopart. Res.* 2000**,** 2, 123-131
